# Bispecific antibody targeting TGF-β and PD-L1 for synergistic cancer immunotherapy

**DOI:** 10.3389/fimmu.2023.1196970

**Published:** 2023-07-13

**Authors:** Tianye Li, Xinrun Wang, Mengke Niu, Mingli Wang, Jianwei Zhou, Kongming Wu, Ming Yi

**Affiliations:** ^1^ Department of Gynecology, The Second Affiliated Hospital, Zhejiang University School of Medicine, Hangzhou, China; ^2^ Zhejiang Provincial Clinical Research Center for Obstetrics and Gynecology, Hangzhou, China; ^3^ Cancer Center, Shanxi Bethune Hospital, Shanxi Academy of Medical Science, Tongji Shanxi Hospital, Third Hospital of Shanxi Medical University, Taiyuan, Shanxi, China; ^4^ Department of Breast Surgery, The First Affiliated Hospital, College of Medicine, Zhejiang University, Hangzhou, China

**Keywords:** cancer immunotherapy, immunotherapy resistance, immune checkpoint inhibitor, bispecific antibody, TGF-β, PD-L1

## Abstract

The PD-1/PD-L1 signaling pathway plays a crucial role in cancer immune evasion, and the use of anti-PD-1/PD-L1 antibodies represents a significant milestone in cancer immunotherapy. However, the low response rate observed in unselected patients and the development of therapeutic resistance remain major obstacles to their clinical application. Accumulating studies showed that overexpressed TGF-β is another immunosuppressive factor apart from traditional immune checkpoints. Actually, the effects of PD-1 and TGF-β pathways are independent and interactive, which work together contributing to the immune evasion of cancer cell. It has been verified that blocking TGF-β and PD-L1 simultaneously could enhance the efficacy of PD-L1 monoclonal antibody and overcome its treatment resistance. Based on the bispecific antibody or fusion protein technology, multiple bispecific and bifunctional antibodies have been developed. In the preclinical and clinical studies, these updated antibodies exhibited potent anti-tumor activity, superior to anti-PD-1/PD-L1 monotherapies. In the review, we summarized the advances of bispecific antibodies targeting TGF-β and PD-L1 in cancer immunotherapy. We believe these next-generation immune checkpoint inhibitors would substantially alter the cancer treatment paradigm, especially in anti-PD-1/PD-L1-resistant patients.

## 1 Background

Programmed cell death 1 (PD-1) is a crucial signaling pathway that inhibits the immune response and helps maintain immune homeostasis (1). However, when overactivated in the tumor microenvironment, this pathway hinders host immune surveillance and clearance of tumor cells (2). Monoclonal antibodies targeting PD-1 or its ligand PD-L1 can restore the activity of exhausted immune cells and enhance the killing effect on tumor cells by blocking this immunosuppressive signaling (3–5). While anti-PD-1/PD-L1 monoclonal antibodies have been clinically approved for treating multiple malignancies and have exhibited promising anti-tumor effects in some patients, the low objective response rate of patients remains a challenge (6). In fact, the cancer-immunity cycle model suggests that in addition to the highly activated PD-1/PD-L1 pathway, multiple factors may become rate-limiting steps that restrict the anti-tumor immune response. Several studies have demonstrated that the activity of the TGF-β pathway in immunotherapy-resistant tumor is significantly increased (7, 8). The highly expressed TGF-β in the tumor microenvironment is also involved in cancer immune escape (9). The immunosuppressive mechanisms of TGF-β and PD-1 pathways are independent and complementary to each other, jointly promoting tumors to escape from host immune surveillance (10).

Highly expressed TGF-β in tumor tissues is primarily secreted by tumor cells and stromal cells. Highly expressed TGF-β not only promotes the epithelial-mesenchymal transition of tumor cells, but also regulates multiple tumor-infiltrating immune cells, leading to the formation of the immunosuppressive tumor microenvironment (11–14). On the one hand, TGF-β suppresses the functions of CD8^+^ T cells and natural killer cells (NK), and on the other hand, upregulates the numbers of regulatory T cells (Treg), M2-like macrophage, and myeloid-derived suppressor cells (MDSCs) (15–18). In addition, it has been confirmed that the high TGF-β tumor microenvironment can improve the activity of tumor-associated fibroblasts (CAFs) and promote the generation of collagen fibers in tumor stroma. The thickened collagen fibers around the tumor tissue are not conducive to immune cell infiltration, eventually forming the immune-excluded tumor type (7). Commonly, this type of tumor does not respond to anti-PD-1/PD-L1 monoclonal antibodies, while blocking TGF-β signaling can significantly reverse the therapeutic resistance of PD-1/PD-L1 blockade therapy and enhance anti-tumor immunotherapy effects (19–22). Theoretically, agents simultaneously blocking TGF-β and PD-1/PD-L1 pathways might have superior anti-tumor activity, relative to anti-PD-1/PD-L1 monoclonal antibodies.

Currently, Merck reported a bifunctional antibody called M7824 that simultaneously blocks PD-L1 and TGF-β (23). M7824 combines PD-L1 antibody with a trap structure targeting TGF-β, acting as a neutralizing receptor for TGF-β. Phase I clinical data indicate that the side effects of M7824 treatment are manageable and therapeutic effects have been observed in multiple types of cancers (24). Later, more bispecific antibodies (BsAbs) such as YM101 and BiTP are developed, which also exhibit potent anti-tumor activities in preclinical and clinical studies (25, 26). BsAbs targeting both PD-1/PD-L1 and TGF-β represent a significant breakthrough and an upgrade to current PD-1/PD-L1 monoclonal antibodies. By synergistically blocking both PD-1/PD-L1 and TGF-β inhibitory signals, these antibodies can effectively promote the transformation from immune-excluded tumors into immune-inflamed tumors. This can improve the efficacy of current PD-1/PD-L1 monoclonal antibodies and broaden their anti-tumor effects spectrum. In this review, we provide a summary of the recent advances in anti-TGF-β/PD-L1 BsAb development. Additionally, we discuss both the advantages and disadvantages of this next-generation immune checkpoint inhibitor.

## 2 The present status of PD-1/PD-L1 blockade

PD-1 is a pivotal immune regulation signal molecule distributed in a wide breadth of immune cells, including DC, T cells, B cells and natural killer cells (NK), and activated monocytes or macrophages (27). PD-1, together with its ligands, PD-L1 and PD-L2, was found to take immunosuppressive effects in the antiviral inflammation and the tumor microenvironment (28, 29). Contemporarily, monoclonal antibodies targeting PD-1/PD-L1 have been widely utilized in clinical settings and exhibited remarkable therapeutic efficacy against various malignancies, particularly advanced and refractory tumors.

### 2.1 The biogenesis and biological pathway of PD-1/PD-L1

PD-1 was initially discovered to play a role in immune suppression of inflammation by serving as a negative feedback regulator. The two ligands, PD-L1 and PD-L2, activate and transmit inhibitory signals to target cells. Relative to PD-L2, PD-L1 is more widely distributed, particularly on tumor cells (27). Recent research has shed light on the elaborated and intriguing expression patterns of PD-L1, which is now known to be present not only on cell membrane, but on various cellular compartments and secreted in extracellular vesicles (30). Specifically, PD-L1 has been found to localize endocellularly on endosomes, the Golgi apparatus’s membrane, and endocytic vesicles. PD-L1 has also been detected in extracellular vesicles, which are involved in intercellular communication and the exchange of biological material between cells. These findings offer fascinating new prospects and possibilities for the development of novel therapeutic strategies targeting PD-L1 in cancer therapy (31). The PD-1/PD-L1 pathway exerts immune regulatory effects via recognition of effector T cells in the inflammatory context, with persistently high expression on activated T cells. Cytokines across the extracellular interval of tissue cells induce and modulate the expression of PD-L1, blunting the activation of T cells, and consequently resulting in immune homeostasis that the immune system eliminates exogenous microbiota while attenuating damage on normal tissue cells simultaneously. The most important inductive cytokine is IFN-γ, mainly derived from Th1 cells (32). PD-1 is persistently expressed at a high level in the circumstance of ongoing inflammation. As a result, persistently high expression of PD-1 triggers T cell exhaustion or inactivation (33).

Mechanistically, the immune system relies on a complex network of interactions between different cells and molecules to mount an effective response against invading pathogens or endogenous abnormalities, including tumor cells. One crucial aspect of this system is the ability of T cells to recognize and respond to antigens presented by other cells. In this bioprocess, MHC II molecules on antigen presentation cells (APCs) or MHC I molecules on all the karyocytes present fragments of antigens on cell membrane, which can be recognized by the T cell receptor (TCR) (34). This interaction activates T cells and triggers a cascade of signaling events that lead to the proliferation and differentiation of T cell, as well as the secretion of cytokines and other effector immune molecules. However, to prevent excessive immune activation and tissue damage, the immune system could dampen or terminate immune responses. Simultaneously with TCR-induced cascade, PD-1 begins to be expressed on the activated T cells. PD-1 and PD-L1 are brought into close proximity to each other in the microscale spatial structure. Subsequently, Immunoreceptor Tyrosine-based Inhibitory Motif (ITIM) and Immunoreceptor Tyrosine-based Switch Motif (ITSM) domain of PD-1 receptor are phosphorylated (35). The phosphorylated domains recruit tyrosine phosphatases, SHP-2 and SHP-1, which are capable of impeding critical factors in TCR signaling (36–38).

Consequently, T cell activation and function are blunted, restraining the degree and duration of the immune response. In addition to signaling repression, PD-1 can also interfere with the recognition of tumor cells by directly dampening the trimeric interaction between the TCR, pMHC, and CD8 molecules (39). This further contributes to immune evasion and tolerance by tumor cells and highlights the importance of PD-1/PD-L1 blockade as a promising immunotherapy strategy for cancer treatment.

### 2.2 The present approved PD-1/PD-L1 blockade therapies

**Figure 1:**
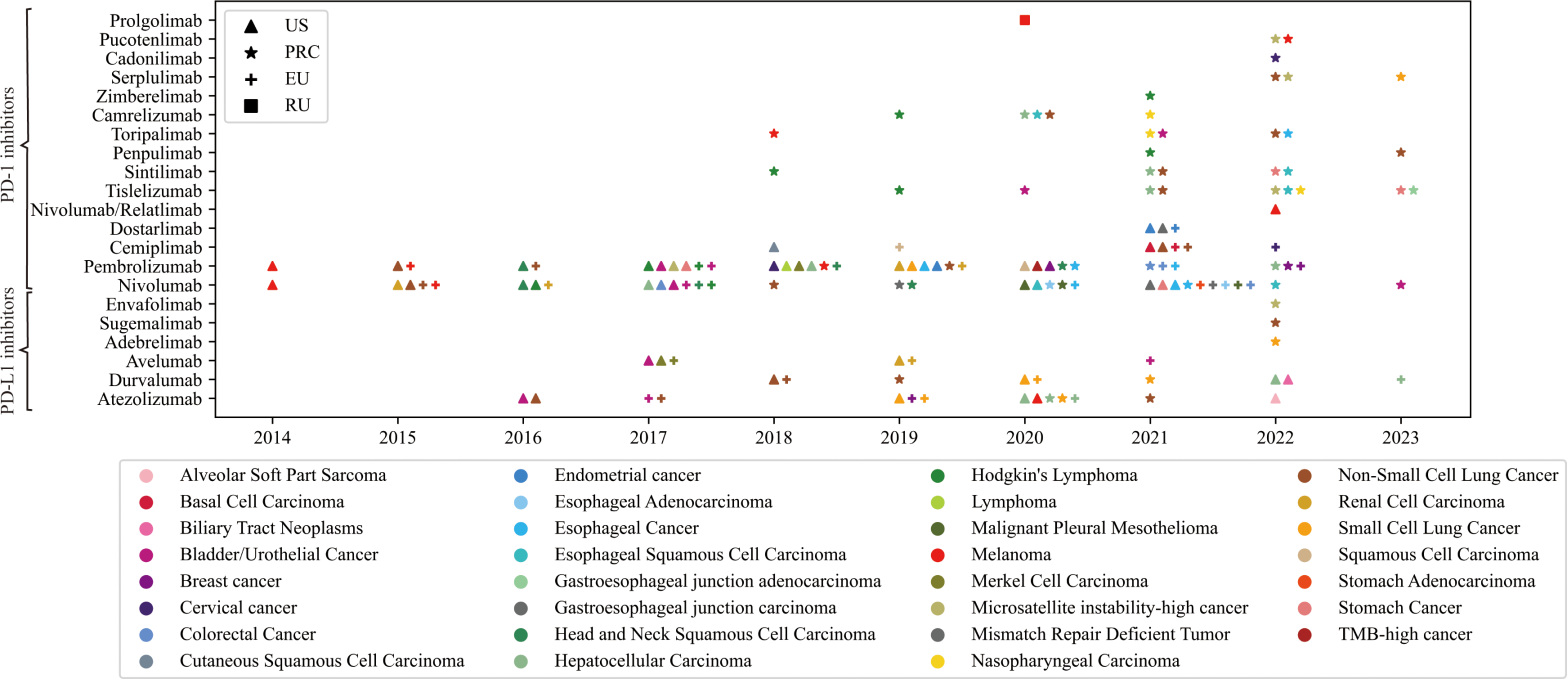
The details of approved PD-1/PD-L1 blockades in the United States of America, European Union, People’s Republic of China, and Russian Federation. Triangle symbol indicates that the corresponding indication of the drug has been approved by the United States Food and Drug Administration (FDA); Stellated symbol indicates that the corresponding indication of the drug has been approved by the National Medical Products Administration (NMPA) of People’s Republic of China. Similarly, other symbols represent drug approvals by corresponding regulatory agencies in European Union and Russian Federation. The abscissa represents the time of the approval. The color of the icon represents the approved indication.

As of March 2023, 21 PD-1/PD-L1 blockade drugs are available worldwide (**Figure 1**) (40–48). Herein, Nivolumab and Pembrolizumab are the most widely used PD-1/PD-L1 blockers and have received approval for the most indications. Notably, Cadonilimab is the first approved anti-PD-1/CTLA-4 BsAb for cervical cancer (44). More drugs targeting these immune checkpoints are in development, and clinical trials are underway to explore new indications for existing drugs. New drugs need to be tested against drugs already on the market to show better efficacy or performance. However, primary or acquired treatment resistance of PD-1/PD-L1 blockades has become an interactive conundrum for both physicians and tumor patients (49). Combination therapy regimens containing PD-1/PD-L1 inhibitors and other agents might be promising strategies for overcoming treatment resistance (50–52).

### 2.3 The challenges of PD-1/PD-L1 blockades in cancer therapy

Although PD-1/PD-L1 blockers have achieved an unprecedented breakthrough in cancer therapy, there have been increasing concerns about their disadvantages and insufficiency in the clinic (53, 54). Besides the adverse events and hyper progression associated with PD-1/PD-L1 blockade therapies (55–57), there is a prevalent concern regarding the limited response rate among cancer patients (58). Commonly, the therapeutic effect of anti-PD-1/PD-L1 antibodies mainly depends on the PD-L1 expression status, such as tumor proportion score (TPS), combined positive score (CPS), and immune cell proportion score (IPS) (59). However, a universe evaluation criterion is absent (60). Moreover, recent investigations suggest that single PD-L1 status is not a reliable indicator of predicting the response of patients to PD-1/PD-L1 blockade (61). Nevertheless, for patients without sufficient histopathologic evidence, the response rate to PD-1/PD-L1 blockades is less than 17% (62). Other responding assessing systems intervene to mount the accuracy, which hinges on the genomic instability, which is defined by tumor mutational burden (TMB) and microsatellite instable (MSI) (63–65). However, there are more or less deficiencies with these systems (66). The low response is partially attributed to the discrepancies in immune cell infiltration phenotype. In theory, the tumor immune microenvironment is classified histopathological as three phenotypes, inflamed, immune-excluded, and immune-desert, respectively. They are inextricably intertwined with immune cytokines level, which includes IFN-γ and TGF-β, fatty acid metabolism, neuroendocrine features, and EMT phenotype (67). Only the first tumor immune type benefits from the immune checkpoint inhibitors (68), but the proportion is less than 50% (67). The immune-excluded type takes up 30 to 50 percent of colorectal and ovarian cancer (69, 70). Herein, TGF-β exerts essential effects for hindrance of immune surveillance and tumor elimination in the immune-excluded type (7). The exploitation of an immunotherapeutic strategy that combines PD-1/PD-L1 blockades and TGF-β inhibiting or trap medicine is a promising direction.

## 3 The effects of TGF-β on anti-tumor immunity

### 3.1 TGF-β signaling

TGF-β superfamily consists of more than 40 members, mainly classified as four subtypes, the TGF-β subtype, the bone morphogenetic protein-growth differentiation factor (BMP-GDM), activin-inhibin-nodal and others, orchestrates in the biological processes of carcinoma initiation, progression, and immune elimination (71–77). TGF-β, the most classic subtype, is a highly conserved and distributed breadth of the organism in the mammal. Three isoforms, TGF-β1, β2, and β3, are highly conserved with 80% of the same amino sequence, despite being encoded by separate genes. However, they still exhibit slight discrepancies in structural and bio-functional aspects, which can be recapitulated that TGF-β1 is more tendentious to immune regulation (78). It is secreted into the extracellular matrix, initially exists in an inactive form of latent precursor, and readily exerts biological functions in the tumor microenvironment via autocrine and paracrine (79). TGF-β is transcribed into a polypeptide, which is then cleaved by the Furin proteinase into two subunits. These subunits are further reassembled into an inactivated form that is secreted out of the cells. The extracellular inactivated TGF-β exists as a large complex, with the dimeric regulatory subunit called the long latency-associated peptide (LAP) forming the peripheral compound. The initial segment of LAP is a short signal peptide called the arginyl-glycyl-aspartic acid site (RGD). The bioactive catalytic subunit is ensconced internally, noncovalently combined with and wrapped around by the dimeric LAPs (72). In most cases, the cage-like complex could anchor via a disulfide bond to a compound of extracellular matrix, namely latent TGFβ-binding protein (LTBP), for stabilization. Additionally, the complex could also bind to transmembrane milieu proteins, particularly glycoprotein A repetitions predominant (GARP) on Treg and negative regulator of reactive oxygen species (NRROS) on microglia or macrophage (80–82). In the extracellular space, physical and chemical perturbance or serine protease would cleave and separate the dimeric LAPs from the complex to release the bioactive subunit. But integrin complexes capable of transmitting force derived from cytoskeleton across the cytomembrane, play the most prominent activated executor role (83). β-αv integrin heterodimer, particularly, recognizes the RGD motif, further interdigitates with the LAP, and consequently, changes autologous conformation. The allosterism tightens and gradually tears the “sleeve” of the latent TGF-β off and exposes the internal bioavailable TGF-β (83). Nevertheless, a unique kind of integrin is able to transduce the signaling without tearing LAP off and releasing the internal bioactive TGF-β (84). The free activated TGF-β or integrin αvβ8-latent TGF-β complex is capable of attaching to their three isoforms of the specific receptor, namely TGFBR1, 2, and 3, in divergent degrees of affinity (85). TGFBR3 doesn’t possess kinase activity like the others but can bind with all types of TGF-β with a high affinity therein. Therefore, it was previously believed to sequester and hinder the redundant TGF-β signaling (86). Recent studies recover its crucial roles in cellular signaling transduction in TGF-β dependent or independent manners (87, 88).

Bioavailable TGF-β could bind with TGFBR2 on the plasm membrane of specific cells. TGFBR1, subsequently, is recruited by the signaling complex, and together companies into a transmembrane heterotetrameric signaling complex. The endo-domain of the allosteric complex is phosphorylated. Then, the signaling cascade is triggered off. There are two signaling processes after the TGF-β-TGFBR1/2 tetrameric complex phosphorylated (10).

#### 3.1.1 Canonical downstream signaling pathway

The Smad2/3 are firstly recruited by the transmembrane signaling and ulteriorly phosphorylated. The phosphorylation induces the binding of Smad4. Then, phosphorylated-Smad2/3/4 (Phospho-Smad complex) is assembled and translocates into the nucleus. As a result, the ultimate signaling complex induces downstream alterations in gene expression (89).

#### 3.1.2 Noncanonical downstream signaling pathway

The redundantly initial transmembrane signaling could cross the recognition of Smad2/3, and directly activate the downstream signaling, such as phosphoinositide‐3‐kinase‐serine/threonine kinase (PI3K-AKT) and mitogen‐activated protein kinase (MAPK). The PI3K-AKT and MAPK signaling ulteriorly cause respective downstream cascades, which regulate the physiologic and pathologic alterations (90).

### 3.2 TGF-β signaling around the development of cancer

**Figure 2:**
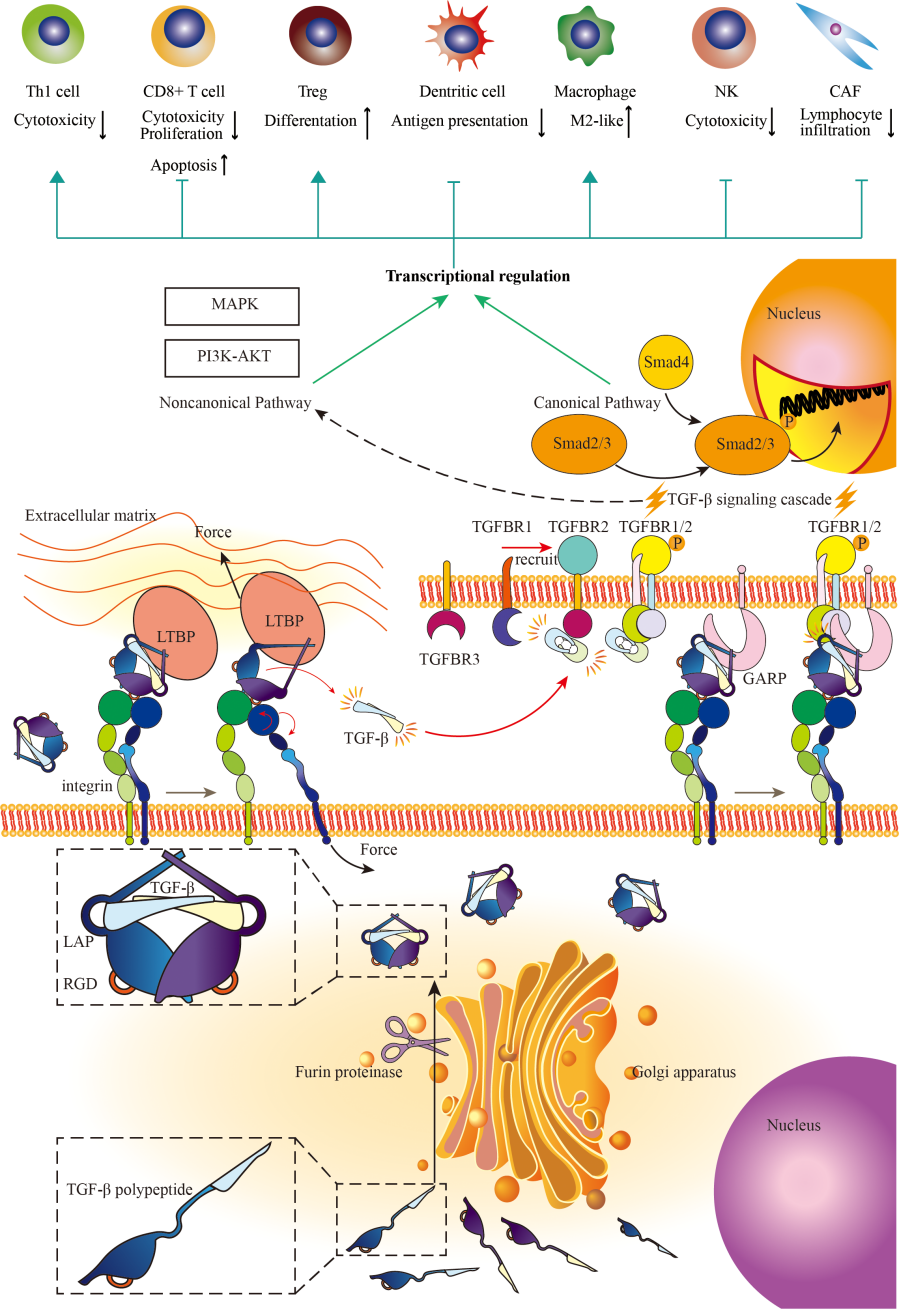
The mechanisms of TGF-β signaling and the immunosuppressive effects of TGF-β signaling on the immune response. Cells with yellow nuclei refer to immune cells and CAFs regulated by TGF-β signaling pathway. Cells with purple nuclei refer to TGF-β-producing cells (mainly secreted by fibroblast). TGF-β is a widely distributed signaling molecule involved in the regulation of almost all kinds of immune cells in the tumor microenvironment. On the one hand, TGF-β limits the functions of T cell, NK, and dendritic cell (DC). On the other hand, TGF-β promotes the differentiation towards Treg and M2-like macrophage. Besides, TGF-β enhances cancer-associated fibroblast (CAF) to generate collagen, which hampers immune cell infiltration. Adapted from Yi et al, 2022 (10).

The smoldering cancer-related inflammation (CRI) accompanied by the development of cancer, with multitudinous kinds of immune cells and immune regulatory cytokines and chemokines pervading across mesenchyme is the persistent intrinsic characteristic of the tumor microenvironment (91). TGF-β, as a pleiotropic cytokine, exerts nuanced, complicated, and even contradictory biological regulatory functions with the development of cancer. In general, TGF-β stimulates the proliferation, transformation, and motility of mesenchyme-originated cells, while inhibiting proliferation and promoting differentiation of epithelium-originated cells and hemopoietic cells (92, 93). In the physiologic condition and early stage of cancer, TGF-β across mesenchyme delicately induces cell cycle arrest and inhibits cell proliferation through the canonical pathway. In the progression of malignancy, the loss of function mutations across the TGF-β pathway and rewiring TGF-β bioprocess make TGF-β a mutineer against tumor suppression signal network, to elicit tumor unconfinedly growing (94). The stimulation of TGF-β, the other cytokines, and chemokines in the tumor microenvironment transforms normal fibroblasts into CAF (95, 96). CAF can prompt tumor progression versatilely (97). In this condition, the tumor cells and cancer-associated fibroblasts excessively secret the amount of TGF-β to the extracellular matrix (97, 98). TGF-β also induces tumor cell EMT, a critical biologic process for migration and invasion, and biological features robustly associated with metastasis (99, 100). Besides, TGF-β can enhance angiogenesis which is beneficial to tumor growth and metastasis through either intracellular pathways or indirectly mediating EMT (101, 102). Apart from the aforementioned direct effects, TGF-β also assumes the paramount role in tumor immunity, indirectly influencing tumor cells throughout the tumor initiation and progression (**Figure 2**).

#### 3.2.1 TGF-β signaling in the tumor immune system

TGF-β is a widely distributed signaling molecule involved in the regulation of almost all kinds of immune cells in the tumor microenvironment. Foxp3 positive Treg, particularly terminally differentiated effector Treg cells, plays a crucial role in tumor immune evasion, suppressing immune recognition and diminishment and ensuring tumor development and metastasis (103). With the development of genetic tracing and multiple-colors flow cytometer analysis, the lineage derivation of Treg has been demonstrated as dual origins, thymic Treg (tTreg) or natural Treg (nTreg), and peripheral Treg (pTreg) or alias-induced Treg (iTreg) (104–106). tTreg, which constitutes 80% of the total Treg repertoire, is derived from CD4-positive T cells that are induced by moderately robust co-stimulation of the TCR and a series of soluble cytokines, such as interleukin-2 (IL-2), IL-7, and IL-15, secreted by other autoreactive immunocytes in the thymus medulla. These cells express Foxp3 more stably and strongly, which is the major component of immune regulation (107–109). The pTreg, despite minor proportion, plays a comprehensive and cryptic role in local immune regulation and immune homeostasis in a Foxp3-independent way (16, 110). TGF-β plays nuanced, complicated, and pleiotropic roles in Tregs of both origins. In thymus medulla, it is highly enriched. The activated TGF-β participates in promoting the differentiation of Treg and the negative selection of neonatal T cells (108). Nevertheless, it is reported when TGF-β signaling is depleted, medullary thymic epithelial cells are stimulated, acting as “caregivers” of Treg cells and ultimately increasing the number of Treg cells (111). It suggests the multiplicity of TGF-β signaling on tTreg.

In the induction of pTreg, TGF-β accompanied with other immune regulatory signals, is capable of boosting Foxp3 expression on mature CD4 positive T cells in the peripheral. In the tumor microenvironment, excessive TGF-β from tumor cells and CAF not only suppresses the proliferation of other normal epithelial cells and conventional immune cells, but facilitates the transformation of pTreg (11). An aforesaid TGF-β anchored transmembrane milieu protein, GARP, is located on Treg, which is essential to extracellular latent TGF-β stabilization. Besides, the GARP on Treg could interact with integrin αvβ8, which also widely distributes on the Treg (112, 113). Both transmembrane proteins orchestrate more efficiently in the signaling transduction on Treg without breaking latent TGF-β off (113). Therefore, the increased pTreg in the local tumor context accelerates the transmission of TGF-β through the GARP pathway. To summarize, TGF-β prompts the activation of Treg of both origins, through whose pathway cancer cells trigger immune evasion and immunotherapy resistance.

The understanding of tumor-infiltrating B cells and their regulatory molecules remains vague. A regulatory type of B cells is identified as IgA positive and capable of inhibiting CD8^+^ T cell activation in colorectal tumors (114). Breg can also secret TGF-β, contributing to the apoptosis of effector T cells in the tumor microenvironment (115).

TGF-β can inhibit immunological surveillance and conventional effector immune cells in the tumor-developing stage. On the one hand, TGF-β directly dampens immunological surveillance by targeting cytotoxic lymphocytes (CTL) (15). On the other hand, it can prevent mature inflammation dendritic cells (DC) infiltration and induces the tolerogenic DC to indirectly deceive the surveillance (116, 117). Although tumor antigen bypasses or breaks through the first immunosuppression barrier to prime and activate the antigen immunity, TGF-β can suppress igniting CRI by decreasing total effector immune cells repertoire by reducing IL-2 secreted (118). Moreover, it precludes the transformation from naïve T cells to Th1/Th2 cells (119–121), but prompts the transformation to anti-inflammation Th17 cells (122, 123). In the natural immunity of tumor context, TGF-β polarizes tumor-associated macrophages to M2 (124), and reprogram tumor-associated neutrophil (125), both of which are detrimental to tumor immune elimination (126). Additionally, TGF-β inhibits the activation and functions of natural killer cells by suppressing the mTOR pathway (81, 127).

## 4 Advances of BsAb

BsAb is a powerful tool for improving the limited sensitivity and effectiveness of traditional antibodies in immunotherapy (128, 129). BsAb can broaden the range of applications by targeting two different molecules and enhancing the anti-tumor effects (130–132). There are two primary types of BsAbs, depending on the presence of the Fc region. The first type is the Fc-containing BsAb, also known as the IgG-like molecule. This type of antibody exerts Fc-mediated effects, such as antibody-dependent cellular cytotoxicity or phagocytosis, and complement-dependent cytotoxicity (133, 134). IgG-like molecules also have a longer half-life than fragment-based molecules (135). Alternatively, the antigen-binding site can be directly connected by a peptide, which lacks the Fc region, known as the fragment-based molecule. This type of antibody has demonstrated flexibility in targeting tumor cells. Additionally, fragment-based molecules have promising potential to develop multi-specific antibodies (136). BsAbs can be developed using one of three methods: genetic or protein engineering, chemical conjugation, and quadroma (137). With these manufacturing methods, a vast array of BsAbs has been developed and tested in clinical trials.

The targeted antigens of BsAb are diverse. To summarize, the main targets include EpCAM, CEA, PSMA, ErbB, GPC3, immune checkpoints like PD-1 and CTLA-4, DLL4, and VEGF (138). The therapeutic effect of BsAb mainly regulates tumor immune response, which can be divided into two parts: immune cell redirection and anti-tumor immunity enhancement. Physiologically, immune cells, especially CD8^+^ T cells, detect and kill potential tumor cells. During tumorigenesis, multiple dysfunctions of T cells result in cancer immune escape (139).

Some BsAbs have two types of binding antigens: a specific tumor-associated antigen (TAA) and an extracellular CD3 subunit located on the T cell surface. This kind of antibody is called T cell-engaging BsAbs (TCE). Thus, TAAs direct T cells to targeted tumor cells. TCE was first introduced in 1985 and has rapidly developed at the beginning of the 21st century (140). Catumaxomab and blinatumomab are representatives of TCE. The TAAs of the two antibodies are EpCAM and CD19 (141, 142). Additionally, other TAAs such as CD20, CEA, gpA33, EGFR variant III, PSMA, MUC-1, glypican-3, P-cadherin, B7-H3, and even intracellular antigens can be TAA of TCE (143–151). Another mechanism of BsAb is anti-tumor immunity enhancement. To achieve this effect, this kind of antibody mainly blocks immune checkpoints. The blockade of innate immune checkpoints like CD47 can disrupt the antiphagocytic signals expressed by tumor cells and enhance phagocytosis by macrophages (152–154). Immune checkpoints of T cell activity can also be blocked to enhance adaptive immunity. PD-1/PD-L1, CTLA-4, LAG3, and TIM3 are receptors of coinhibitory immune checkpoints. The blockade of these molecules resuscitates the function of tumor-infiltrating T cells in various kinds of cancers in 10%-30% of patients (155). Molecules in the TNF receptor superfamily and glucocorticoid-induced TNFR-related protein are receptors of costimulatory immune checkpoints. Using an agonist to activate the receptors can reverse the suppression of CTL and promote tumor cell death (156). The combination of blockade of coinhibitory molecules and activation of costimulatory molecules also achieves promising anti-tumor effects *in vivo* (40, 157, 158). The immune cell redirection and anti-tumor immunity enhancement can also be combined to achieve a robust anti-tumor effect *in vivo* (159).

However, there are some adverse effects of BsAb that cannot be eliminated at present. Since BsAb primarily regulates the immune response through signaling pathways and cytokines, the most common adverse effect is cytokine release syndrome (CRS) (160, 161). CRS is caused by a large number of cytokines, such as IFN-γ secreted by activated T cells (162, 163). They can suppress the overstimulated inflammatory reactions without significantly reducing the anti-tumor effect (147). Cytokine receptor antagonism can block the receptors of overexpressed cytokines and relieve the symptoms of CRS (164, 165). However, to prevent the potential immune suppressive effect of corticosteroids, small molecule inhibitors have been developed. This type of inhibitor specifically targets the signaling molecules involved in CRS induction. Since most cytokines promote inflammation via the JAK/STAT pathway in CRS, preclinical studies of JAK1/2 inhibitors have shown promising efficacy in preventing CRS (166). In addition, Bruton’s tyrosine kinase inhibitors (BTK) can directly bind to B cells to reduce the overexpression of cytokines caused by BTK signaling, preventing CRS without affecting anti-tumor efficacy (167).

## 5 Anti-TGF-β/PD-L1 bsAb (including bifunctional protein)

Given the high expression levels and specific enrichment of both PD-L1 and TGF-β in the TME, reagents that target them simultaneously could provide more precise targeting of cancerous lesions while sparing normal tissues. As a result, bispecific antibodies (BsAbs) may accumulate in the TME, reducing side effects and improving tumoral precision therapy.

### 5.1 YM101 and BiTP

**Figure 3:**
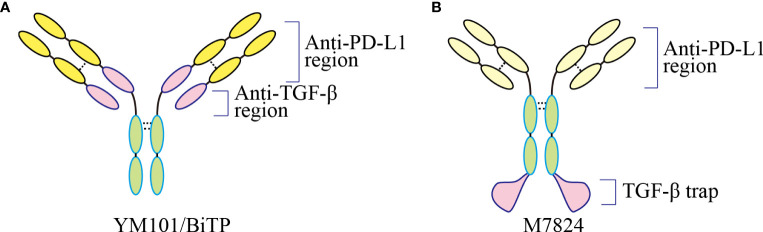
The structure of bispecific and bifunctional antibodies targeting TGF-β and PD-L1. **(A)** The structure of YM101 and BiTP. YM101 and BiTP contain anti-TGF-β and anti-PD-L1 domains in Fab region. **(B)** The structure of M7824. M7824 contains an anti-PD-L1 domain in Fab region and a TGF-β trap in Fc region. Adapted from Yi et al, 2022 (40).

YM101 is the world’s first publicly reported anti-TGF-β/PD-L1 bsAb (25). Although fusion protein M7824 has been published before, it is the first time to target these two molecules by bsAb technology. YM101 is the first molecule developed based on the Check-BODY™ technology platform (**Figure 3**) (25). The results showed that YM101 could effectively antagonize the biological effects of TGF-β and PD-1/PD-L1 pathways. In addition, *in vivo* experiments showed that the anti-tumor activity of YM101 was superior to that of anti-TGF-β and anti-PD-L1 monotherapies. Investigations into the TME found that YM101 promoted the formation of inflamed tumor: increased the number and activity of TIL and DC, and increased the ratio of M1/M2 macrophages. Additionally, hyperactive TGF-β signaling in CAF leads to thickened peritumoral collagen, which hampers immune cell infiltration and limits the efficacy of anti-PD-L1. However, YM101 suppressed the functions of CAFs and undermined the peritumoral barrier by neutralizing TGF-β in the TME. As a result, YM101 promoted T cell infiltration and relieved anti-PD-L1 resistance (25). Moreover, the combination therapy of Mn^2+^ and YM101 has been shown to have a synergistic anti-tumor effect, effectively reversing immunotherapy resistance in non-inflamed tumors (168). It has been validated that that Mn^2+^ activates the STING pathway, promotes DC maturation, and cooperates with YM101 to promote T cell activation. Moreover, in multiple mouse tumor models, the combination of Mn^2+^ and YM101 treatment has exhibited durable anti-tumor effects and prolonged the survival of tumor-bearing mice (168). Compared to monotherapy, the combination of Mn^2+^ and YM101 has a stronger anti-tumor effect and a broader anti-tumor spectrum. Mechanistically, this strategy (168). Further single-cell transcriptome analysis demonstrated that STING agonist combined with YM101 simultaneously regulates multiple components of anti-tumor immunity, promoting the transition from immune-exclude or immune-desert to inflamed tumors. This novel combined approach has the potential to be a general treatment for both inflamed and non-inflamed tumors (74). Encouraged by the positive preclinical data, the alternative molecule BiTP was constructed for further clinical trials (26). With a similar structure to YM101, BiTP is created by Check-BODY™ as well. The results of murine triple-negative breast cancer models showed that BiTP decreased peritumoral collagen generation and promoted T cell infiltration (26). A phase I clinical trial (NCT05028556) is also on recruiting to explore the optimal dose, efficacy, and safety of BiTP. There has been no observation of serious immune-related adverse events in the trial.

### 5.2 M7824

As a novel bifunctional fusion protein targeting TGF-β and PD-L1, M7824 contains an anti-PD-L1 domain in Fab region and a TGF-β trap in Fc region (23). In murine cancer models, M7824 showed potent anti-tumor efficacy and significantly prolonged the survival of tumor-bearing mice (23). Further investigations showed that M7824 substantially reshaped the tumor immune microenvironment: upregulating the numbers and activities of tumor-killing effectors and decreasing the ratio of immunosuppressive subsets such as MDSC, M2-like macrophage, and Treg (23). Also, M7824 led to tumor matrix remodeling, which might contribute to improved immune cell infiltration (23). Notably, preclinical data indicated that radiotherapy or chemotherapy might enhance the anti-tumor effect of M7824 (23, 169). The successful *in-vivo* studies and animal studies inspire researchers to conduct clinical trials associated with M7824 as listed in **Table 1**. In the phase 1 trial NCT02517398, the response rate was 87.5% in patients with PD-L1 high NSCLC (170). Up to now, M7824 has undergone 19 clinical trials, with 4 completed, 1 actively not recruiting, 12 terminated and 2 withdrawn, according to the ClinicalTrials database (**Supplementary File: Table S1**).

**Table 1:** Clinical trials of M7824.

NCT number	Cancer type	Phase	Primary Measure Outcomes	Status
NCT03833661	Biliary tract cancer, Cholangiocarcinoma, Gallbladder cancer	2	ORR	Completed
NCT03524170	Breast cancer	1	Safety	Completed
NCT03840915	NSCLC	1/2	Safety	Completed
NCT02699515	Solid tumors	1	Safety	Completed
NCT04066491	Biliary tract cancer, Cholangiocarcinoma, Gallbladder cancer	2/3	Safety and OS	Completed
NCT02517398	Solid tumors	1	Safety	Completed
NCT04489940	TNBC	2	ORR	Completed
NCT04246489	Uterine cervical neoplasms	2	ORR	Completed
NCT04220775	HNSCC	1/2	Safety and PFS	Completed
NCT04551950	Cervical cancer	1	Safety	Completed
NCT04501094	Urothelial cancer	2	ORR	Terminated
NCT03840902	NSCLC	2	PFS	Terminated
NCT03451773	Pancreatic cancer	1/2	Safety and ORR	Terminated
NCT04327986	Pancreatic cancer	1/2	PR2D, Safety, and ORR	Terminated
NCT04560686	NSCLC	2	ORR	Terminated
NCT04428047	HNSCC	2	ORR	Terminated
NCT04727541	Cholangiocarcinoma	2	ORR	Terminated
NCT04971187	NSCLC	2	ORR and PFS	Terminated
NCT04417660	Thymic cancer	2	ORR	Recruiting
NCT05005429	Mesothelioma and lung cancer	2	PFS	Recruiting
NCT04303117	Kaposi sarcoma	1/2	Safety	Recruiting
NCT04432597	HPV+ cancer	1/2	Safety, PR2D, and CD3+ TIL	Recruiting
NCT03554473	SCLC	1/2	ORR	Recruiting
NCT03493945	Prostate cancer	1/2	Clinical benefit	Recruiting
NCT05012098	Olfactory neuroblastoma	2	ORR	Recruiting
NCT03315871	Prostate cancer	2	PSA	Recruiting
NCT04708470	Solid tumors	1/2	ORR and PR2D	Recruiting
NCT03427411	Solid tumors	2	ORR	Active, not recruiting
NCT03631706	NSCLC	3	PFS and OS	Active, not recruiting
NCT03436563	Colorectal cancer, MSI-H solid tumors	1/2	ORR, ctDNA	Active, not recruiting
NCT05061823	Lung cancer	3	Safety	Active, not recruiting
NCT04247282	Head and neck cancer	1/2	ORR	Active, not recruiting
NCT04574583	Solid tumors	1/2	ORR	Active, not recruiting
NCT04491955	Small bowel cancer, colorectal cancer	2	ORR	Active, not recruiting
NCT04287868	Solid tumors	1/2	ORR	Active, not recruiting
NCT04789668	Solid tumors	1/2	ORR, PR2D, Safety, and OS	Active, not recruiting
NCT04396535	NSCLC	2	PFS	Active, not recruiting

ORR, objective response rate; NSCLC, non-small cell lung cancer; SCLC, small cell lung cancer; TNBC, triple negative breast cancer; OS, overall survival; HNSCC, head and neck squamous cell carcinoma; PFS, progression-free survival; PR2D, recommended phase II dose; TIL, tumor-infiltrating lymphocyte; MSI-H, microsatellite instability-high; ctDNA, circulating tumor DNA.

### 5.3 Other novel antibodies

The triumph of M7824 has stimulated the exploration of novel fusion protein endeavors, among which is SHR-1701, a monoclonal anti-PD-L1 domain fused with an N-terminal-truncated domain of TGFβRII that bears a resemblance to M7824 in structure (171). The linked TGFβRII domain serves as a trap and neutralizes TGF-β in the tumor microenvironment, while the Fab segment of the antibody blocks PD-L1. This dual blockade overcomes anti-PD-1 resistance in murine tumor models (172). In advanced tumors, SHR-1701 showed anti-tumor activity with objective response rate (ORR) of over 20% (173). In recurrent and metastatic cervical cancer, the ORR of SHR-1701 reached 15.6% (174). Also, fusion protein BR102 contains anti-PD-L1 antibody and TGFβRII ectodomain (175). Further animal studies confirmed the anti-tumor activity of BR102 in murine tumor models (176).

## 6 Perspective and Conclusion

For advanced cancers, TGF-β changes from a tumor suppressor to a tumor promoter. In cancer immunology, TGF-β substantially undermines immune surveillance and immune clearance by limiting the activities of antigen-presenting cells and cytotoxic T cells. Therefore, TGF-β blockade is a promising approach to improve immunotherapy performance. Although the enhanced anti-tumor effect of TGF-β and PD-L1 dual blockade has been validated in several clinical studies, the combination therapy of two antibodies indeed complicates grouping in clinical trials.

Based on BsAb or fusion protein technology, multiple BsAbs have been developed, which could simultaneously counteract PD-1 and TGF-β signaling pathways. Commonly, these BsAbs exhibit more potent anti-tumor activities and effectively reshape the immunosuppressive microenvironment. Notably, the therapeutic effect of anti-TGF-β/PD-L1 BsAb is even superior to anti-TGF-β plus anti-PD-L1 treatment, which might be attributed to the high tumor specificity brought by BsAb structure. We believe anti-TGF-β/PD-L1 BsAb has a significant advantage in treatment effect, especially in TGF-β-driven immune-excluded tumors.

However, in a head-to-head phase III clinical study with pembrolizumab, M7824 failed to achieve the expected endpoints in patients with non-small cell lung cancer and cholangiocarcinoma. Although the reasons for the large discrepancy between the results of the phase III trial and the phase I trial have not been published, the lack of precise molecular markers to select suitable patients may be one reason for the failure of the phase III trial. For immune-desert tumors, both TGF-β and PD-1 pathways are not primary rheostats for the cancer-immunity cycle. In this case, combination therapy with agents stimulating antigen release or improving antigen-presenting cell functions is essential to overcome immunotherapy resistance. It has been confirmed that anti-TGF-β/PD-L1 BsAb combined with STING agonist effectively conquers anti-PD-1/PD-L1 resistance in immune-desert and immune-exclude tumors. Hereto, anti-TGF-β/PD-L1 BsAb-involved combination therapy might effectively broaden the anti-tumor spectrum of immunotherapy in the future.

## Author contributions

TL and XW performed the selection of literature, drafted the manuscript and prepared the figures. MN, MW, and JZ collected the related references and participated in discussion. MY and KW designed this review and revised the manuscript. All authors contributed to this manuscript. All authors read and approved the final manuscript.
