# Supplementary material for: Bispecific antibody targeting TGF-β and PD-L1 for synergistic cancer immunotherapy
Source: Front Immunol. 2023 Jul 13;14:1196970. doi: 10.3389/fimmu.2023.1196970 (PMC10373067; doi:10.3389/fimmu.2023.1196970)
Supplement: Supplementary file 1 [file Table_1.docx]

**Table S1:** The results of M7824 clinical trails or reasons of termination or withdrawn.

| NCT numbers | Study name | Status | Results of clinical trails/The reasons of termination or withdrawn |
| --- | --- | --- | --- |
| NCT03707587 | Recurrent Respiratory Papillomatosis | Completed | Partial Response: 14.3% |
| NCT03427411 | HPV Associated Malignancies | Completed | ORR of Participants Naïve to Checkpoint Inhibition: 31%. ORR of Participants Refractory to Checkpoint Inhibition: 7.7%. |
| NCT03833661 | Biliary Tract Cancer | Completed | ORR: 10.1%. |
| NCT03631706 | NSCLC | Active, not recruiting | PFS of M7824: 7.0 months; OS of M7824: 21.1 months. |
| NCT04066491 | Biliary Tract Cancer | Completed | Number of Participants Who Experienced Dose Limiting Toxicities: 0; OS for M7824 + Gemcitabine + Cisplatin: 11.5 months. |
| NCT04501094 | Urothelial Carcinoma | Terminated | Low accrual and Safety concerns. |
| NCT04297748 | NSCLC | Terminated | Efficacy finding. |
| NCT03451773 | Adenocarcinoma of the Pancreas | Terminated | Safety concerns. |
| NCT04327986 | Advanced Pancreas Cancer | Terminated | Low accrual. |
| NCT03840902 | NSCLC | Terminated | Low likelihood of achieving superiority in the efficacy endpoints |
| NCT03579472 | Triple Negative Breast Cancer | Terminated | Due to the fact that the sponsor decided not to move forward with the development of M7824. |
| NCT04296942 | Breast Cancer | Terminated | Slow accrual and safety concerns. |
| NCT04489940 | Triple Negative Breast Cancer | Terminated | Low probability of success. |
| NCT04560686 | NSCLC | Terminated | Per PI Request |
| NCT04428047 | Head and Neck Squamous Cell Carcinoma | Terminated | Safety concerns |
| NCT04727541 | Biliary Tract Cancer | Terminated | Unlikely to meet the primary endpoint of OS. |
| NCT04971187 | NSCLC | Terminated | PI Request |
| NCT04756505 | Breast Cancer | Withdrawn | Per Sponsor Request-no longer manufacturing the study drug |
| NCT04648826 | Sarcomas, Germ Cell Tumors, or Epithelial Malignancies | Withdrawn | Due to notification from EMD Serono, for Bintrafusp alfa (M7824) regarding an observed increase in the frequently of early progression and death. |

Note: NSCLC: Non-small cell lung cancer; ORR: Objective response rate; TRAE: Treatment-related adverse events; DOR: Duration of response; IDMC: Independent Data Monitoring Committee; PI: principle investigator.
